# Biomechanics in Removable Partial Dentures: A Literature Review of FEA-Based Studies

**DOI:** 10.1155/2021/5699962

**Published:** 2021-08-26

**Authors:** Mohammed A. Mousa, Johari Yap Abdullah, Nafij B. Jamayet, Mohamed I. El-Anwar, Kiran Kumar Ganji, Mohammad Khursheed Alam, Adam Husein

**Affiliations:** ^1^Prosthodontic Unit, School of Dental Sciences, Universiti Sains Malaysia, 16150 Kubang Kerian, Kelantan, Malaysia; ^2^Department of Prosthetic Dental Sciences, College of Dentistry, Jouf University, Sakakah, Jouf, Saudi Arabia; ^3^Craniofacial Imaging and Additive Manufacturing Laboratory, School of Dental Sciences, Universiti Sains Malaysia, 16150 Kubang Kerian, Kelantan, Malaysia; ^4^Division of Restorative Dentistry, School of Dentistry, International Medical University, Bukit Jalil, Jalan Jalil Perkasa 19, 57000 Kuala Lumpur, Malaysia; ^5^Mechanical Engineering Department, National Research Centre, Giza, Egypt; ^6^Department of Preventive Dentistry, College of Dentistry, Jouf University, Sakakah, Saudi Arabia

## Abstract

The present study was aimed at reviewing the studies that used finite element analysis (FEA) to estimate the biomechanical stress arising in removable partial dentures (RPDs) and how to optimize it. A literature survey was conducted for the English full-text articles, which used only FEA to estimate the stress developed in RPDs from Jan 2000 to May 2021. In RPDs, the retaining and supporting structures are subjected to dynamic loads during insertion and removal of the prosthesis as well as during function. The majority of stresses in free-end saddle (FES) RPDs are concentrated in the shoulder of the clasp, the horizontal curvature of the gingival approaching clasp, and the part of the major connector next to terminal abutments. Clasps fabricated from flexible materials were beneficial to eliminate the stress in the abutment, while rigid materials were preferred for major connectors to eliminate the displacement of the prosthesis. In implant-assisted RPD, the implant receive the majority of the load, thereby reducing the stress on the abutment and reducing the displacement of the prosthesis. The amount of stress in the implant decreases with zero or minimal angulation, using long and wide implants, and when the implants are placed in the first molar area.

## 1. Introduction

The main objective of removable partial dentures (RPDs) is to provide prosthetic rehabilitation of missing teeth and associated structures, with avoidance of further loss of remaining teeth. RPDs are indicated (in terms of aesthetic and masticatory efficiency) when the edentulous span is extensive, horizontally and vertically, to be treated with conventional fixed dental restoration because of the excessive resorption that may happen following extraction [1, 2]. RPDs are still considered a cost-effective treatment option in partially edentulous patients, compared to fixed and implant-retained restorations [3]. Although there was no worldwide meta-analysis report about the prevalence of patients wearing RPD, up to the authors' knowledge, there was an agreement that the number of partial edentulism is increasing in the United States and the United Kingdom [4–6], with more prevalence in female patients [7]. In Brazil, Kennedy class I was the most prevalent lower edentulism, while Kennedy class III was the most frequent maxillary one [7]. 13% of the United Kingdom population was reported wearing RPDs, while 6% wear complete dentures [8, 9]. As per the mentioned reports, RPDs still provide realistic and predictable treatment options, and therefore, all efforts should be done to design adequate prostheses that serve efficiently with no or minimal damage.

The prosthetic management of partially edentulous patients with RPDs remains to face challenges due to varieties of factors including dental factors, patient's factors, and factors related to the prosthesis itself [10–12]. Components of the prosthesis are subjected to stress and, at the same time, can produce stresses in the supporting structures as well [13]. Abutment teeth, as supporting and retaining structures to the prosthesis, are subjected to stress during function, insertion, and removal of the prosthesis. If this stress exceeded their natural resistance, this may result in resorption in the supporting alveolar bone, loss of the abutment, and, eventually, failure of the prosthesis [14, 15]. In the same way, the free-end saddle prostheses are subjected to stress during function, resulting in bone resorption, loss of the support, and loss of stability of prostheses, which necessitate a frequent replacement [16, 17]. Implant-assisted RPDs showed welcomed treatment modalities compared with the conventional RPDs, in terms of preserving supporting structures, optimizing the retention and stability of the prostheses, improvising the chewing efficiency, and improving the quality of patient life [18–21]. On the other hand, the implant does not show the same tolerance of the natural tooth to the different kinds of occlusal force which, if it exceeded its limit, may result in bone resorption around the implant or even fracture of the implant itself [22, 23]. The occlusal considerations, design of the prosthesis, implant length, diameter, and macro- and micro-surface texture of the implant, bone quantity, and patient factors, play a major role in the survival of implants [24–26].

The biomechanics of oral structures and prosthetic restoration used in dentistry highly influence the long-term success of dental treatment. Therefore, it was crucial to investigate the biomechanical interaction between supporting structures and the overlying prosthesis, in order to control it, to preserve the remaining structures and to maintain the prosthesis working adequately [27, 28].

Measurement of stress in abutment teeth, implants and surrounding structures, and prostheses has been performed using diversities of methods including analytical, numerical, and experimental methods [29]. Experimental methods such as electrical strain gauges can provide point-to-point precise quantitative measurement to the stress distribution in *in vivo* and *in vitro* scenarios [30], while photoelasticity can provide a full-field qualitative measurement of the same kind of stress [31]. Each technique of experimental methods has its advantages and limitations, which make it necessary to use two or more methods to identify the stress and strain in any area of interest [31, 32].

**Figure 1:**
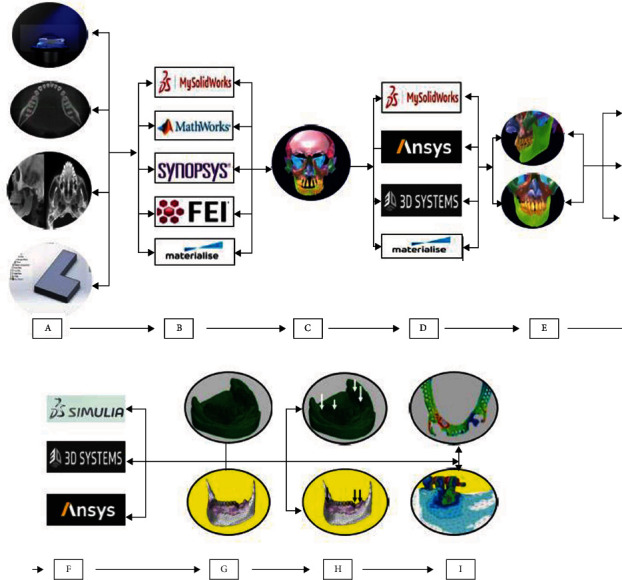
The workflow steps of finite element analysis.

**Figure 2:**
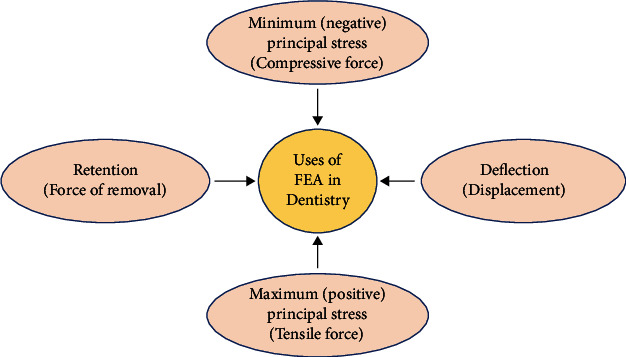


Finite element analysis (FEA), as a numerical method, has been approved as a proficient way of providing qualitative and quantitative mathematical data of the biomechanics of different dental prostheses and their supporting structures [33–37]. The main advantage of FEA is the capability to work with complex situations (or defects) and creating their corresponding prostheses virtually without the need to get ethical approval [38]. FEA can be performed by achieving the personal data from laser scanning, Cone Beam Computerized Tomography (CBCT), Magnetic Resonance Imaging (MRI) or even simulating the design using available computer-aided engineering software. This is followed by customized segmentation, specifying the properties of the materials, getting the model, meshing it, loading that model, and, finally, getting the solution to the problem (Figure 1) [33, 35, 39]. Introduction of nonlinear contact analyses in FEA has solved the soft tissue behavior, problems of sliding, prediction of the deflection and permanent deformation of clasp arms, and the friction phenomena that happen between the prosthesis and abutment teeth and at the proximal contact surfaces between adjacent teeth [40, 41].  Figure 2 shows the problems that FEA can solve in dentistry.

Although the study of biomechanical stress developed in RPDs using FEA is rapidly expanding, there was no broad review in the literature, up to the best of authors' knowledge, concerned with the collection of the stress developed in RPDs and how to minimize it. The purpose of the present review was to identify the distribution of the biomechanical stress in components of RPD and their supporting structures, to elaborate the causes of this stress, and to optimize prostheses design in order to reduce this stress, from the point of perspective of FEA studies. The disparate themes and data make this study unsuited to be in the form of an ordinary systematic review or meta-analysis study.

## 2. Strategy of the Literature Search

This study is a part of a PhD research protocol approved by the Human Research Ethics Committee of Universiti Sains Malaysia (HREC/USM) with JEPeM Code: USM/JEPeM/21030222. An electronic search was conducted by using “Google Scholar, Saudi Digital Library (SDL), PubMed, Scopus, and Web of Science (WOS)” database research tools. The keywords used for the present study were chosen to be more general (“finite element analysis”, “implant-assisted RPD”, and “removable partial denture”) to allow extraction of all relevant data. The initial search was done in “Google Scholar” by the first 3 authors (M.A.M., J.A., N.J.), “SDL” by (M.A.M., K.K.G.), “PubMed and Scopus” by (M.K.A., M.E.), and “WOS” by (J.A., A.H.). The titles and abstracts of the data sources were screened over nearly one month. When an article was found relevant to the objective of our study, its references were screened for further studies that meet the inclusion criteria. The search was done to find answers for the question “what are the factors contributing to the development of the biomechanical stress in RPDs and how to minimize it?” Table 1 shows the inclusion and exclusion criteria of the present review. All picked articles were collected, the required data were extracted, and duplicated articles were excluded from the study. Although there are different software programs, techniques are currently available for performing FEA studies; however, FEA is a numerical process with reproducible data of the same quality.

## 3. Results and Discussion

**Figure 3:**
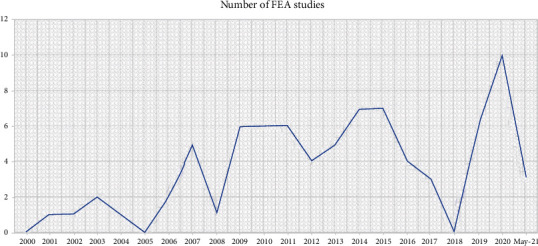
The graph shows the number of FEA studies conducted on RPDs from 2000 to May 2021.

During the selected times, 8258 articles were surveyed. Out of these articles, 8178 were excluded based on screening of the title and abstract (as they do not relate to the objectives of the current review), while 44 articles were finally recruited for this study [17, 21, 36, 42–82]. The initial causes of exclusion of articles were the articles worked on complete, maxillofacial, fixed, and non-FEA methods.  Figure 3 shows the prevalence of conducted English studies that used FEA in RPDs in the last 20 years. It shows an increase in publications of FEA studies in 2020 compared to 2008 and before (except 2018, which showed zero publications). The results were extracted and grouped to identify the targeted problem and how to solve it. Among the selected articles, 14 studies have reported the influence of different retainer designs [44, 55–67], while seven studies measured the influence of different designs and materials of major connectors [66, 69–74], and the studies concerned with implant-assisted RPD were ten articles [17, 43, 55, 76–82]. The results were broad-covering with heterogeneous and disparate data, which made it not compatible to be a meta-analysis or systematic review. For this, a form of narrative review has been chosen for the current research.

There is no disput about the fact that the long-term success of RPDs is directly proportional to the extent of control of various stresses induced by them on the supporting structure. This concept is emphasized by a long history of evaluation of each type of stress and suggesting the optimal design and materials for bringing them to the physiologic limits of the supporting structure [28]. According to the literature, the stress from the RPD components arises from either an accurately designed and fabricated prostheses or prostheses with inaccurate design or fault fabrication. The stress arising from an accurately designed prosthesis is affected by prosthesis (or prosthodontist) factors and patient's factors. The factors relating to the prosthesis include major connector designs, retainer designs, locations of the occlusal rest, properties of denture material, extension of edentulous saddles, and the presence or absence of implant/s. The factors related to the patient include age, ridge shape and form, occlusal force, and type of torque on the prosthesis. The stress arises from inaccurately designed prosthesis including thickness of the framework of the prosthesis, design of the major connector, thickness of the occlusal rest, and selection of the materials [27, 28]. As all FEA studies assumed that the designed prostheses are optimal, regarding the design and materials, and well fitted on their model, the current review focused on the influence of different RPD designs and materials in the development of the stress and how to manage it. To be more convenient to readers, the findings have been categorized under the main titles “Identification areas of stress concentration and deflection in RPDs” and “Factors affecting the biomechanical stress in RPDs”.

### 3.1. Areas of Stress Concentration in RPDs

**Figure 4:**
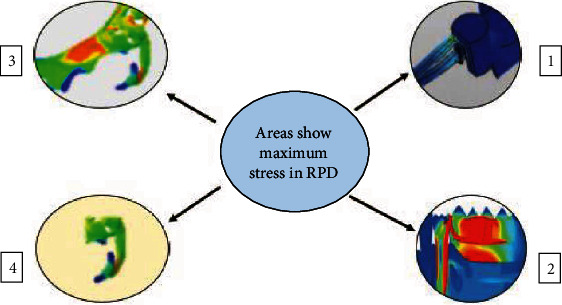
The portions of RPD components that receive the maximum stress.

Although there was a shortage in the literature regarding the identification of stress distribution in tooth-supported RPDs, FES scenarios got much interest regarding this interest. It was found that the terminal abutment shows a concentration of the stress in the apical and distal side [42], while the residual ridge shows the main stress concentration at the occlusal and lingual side when the saddle is short [36], and both of the mesial and distal area when the saddle is long [43]. The most common areas of FES RPD components subjected to stress during function of the prosthesis include; the minor and major connector lingual to the terminal abutment, the horizontal curvature of the gingival approaching clasp [44], and the shoulder of the Aker and back action clasp (Figure 4) [45, 46]. However, the proper design of the prosthesis makes the stress concentration be within the yield strength of the Co-Cr alloy, which results in an extension of the survival rate of the clasp to 5.5 years [45, 46].

In implant-assisted RPDs, the stress is concentrated evenly around the implant if they were fully implant-retained [21]. In the case the scenario was FES RPDs, the stress is mainly concentrated in the mesial side of the implant [49, 50], while the stress in abutments and residual ridges is significantly decreased regardless of the position, length, or width of the implant used [47, 48].

### 3.2. Displacement and Deflection in RPDs

The displacement induced by RPD mainly results from the deflection of the prosthesis, which was affected by mechanical properties of the base materials and the length of the saddle. As the rigidity of the major connector increases, the defection of the denture base materials decreases, while the stress in abutments and implant increases. The displacement of FES RPD is concentrated in the posterior part (distal) of the saddle of the prosthesis. As the saddle length increases, the displacement increases [43, 51]. To minimize the adverse effect of long FES RPDs on supporting structures, implant-assisted RPD would be considered [52, 53]. Upon using implant-assisted RPD, the displacement of the prosthesis significantly decreases regardless of the length, position, width, or inclination of the implant [50, 54].

### 3.3. Factors Affecting the Biomechanical Stress in Conventional RPDs

To overcome adverse effects of biomechanical stress in RPDs and decrease the stress on the supporting structures, a variety of different approaches have been advocated in the FEA literature.

#### 3.3.1. Design of Retention

The retention of conventional RPDs is mainly gained from the adjacent teeth and underlying tissues. There are different types of retainer systems such as occlusal approaching clasps, gingival approaching clasps, rigid and nonrigid attachment systems, telescopic crowns, and implant/s.  Table 2 shows that the studies evaluated the different retainer designs and their influences on the stress and displacement of RPDs.

For bounded saddles, although the circumferential Co-Cr clasp showed the maximum force of removal, maximum rigidity, and highest stability to the prostheses, it exhibits the maximum stress on the abutment teeth [55]. Moreover, the clasp arms are subjected to stress that concentrated at the junction of arms and the body of minor connector, which may result in loss of efficiency or even fracture of the clasp [46]. The magnitude of the stress depends on the depth of undercut, the length of the clasp, and the material of construction [55]. To decrease the stress arising in Co-Cr circumferential clasps, a formula was introduced to optimize the length and width of the clasp. According to this formula, the clasp should be a half-round shape with W2/W1 = 0.6 and *T*/*L* = 0.5 to express the least stress, while W1 is the width of the clasp at the base, W2 is the width of the clasp at the tip, *T* is the thickness, and *L* is the length [56]. More flexible materials were introduced to substitute the Co-Cr materials as well. Among these materials, titanium alloys, gold alloys, polyetheretherketones (PEEK), polyamides, polyoxymethylenes, and acetal resin are examples [55, 57]. Clasps made from polyamides, followed by polyoxymethylenes, were found to produce the least amount of stress on abutment teeth compared to clasps made of Co-Cr and titaniums, regardless of the depth they engage [55]. In the same respect, the clasp made of acetal resin results in less stress when compared with the Co-Cr clasp, despite the retention not being comparable between Co-Cr and acetal resins [57].

In FES RPDs, there are diversities of retainer designs that can be used. Gingival approaching clasps as a most used retainers for FES RPD received the most interest in the literature. When compared with Aker, reverse Aker, and embrasure clasp, the I-bar clasp (of the same material) shows a less distal displacement and stress in PL of abutment teeth when engaged in a 0.01-inch undercut, while the embrasure clasp shows lesser vertical displacement (tissue ward) in the same undercut depth [58]. Reverse Aker brings more load on the main abutment but also shows higher stability and lesser deflection in the prosthesis in the same undercut depth [59]. The RPI system was found to produce stress and at the same was subjected to stress and deformations as well. The stress is concentrated in the neck of the retentive arm (just before the retentive tip) and at the horizontal curvature of the clasp [44, 55]. The magnitude of stress depends on many factors: the thickness and width of clasp arms, the taper and radii of the retentive arm, the shape and curvature of the horizontal approach arm, and the vertical distance between retentive tip and horizontal axis [60, 61]. The most vulnerable area to stress concentration in the RPI system is located in the inner surface of the retentive clasp arm and the area just above the vertical projection of the horizontal arm [62]. To optimize the length and width of the I-bar clasp arm, a thinner and wider arm with a taper of 0.02-0.03 and radius of 2.75-3.00 mm was advocated to reduce the stress in the abutment tooth [61]. It was found that the optimal length of horizontal and vertical arms should not exceed 6 mm to optimize the biomechanical stress within the clasp [60]. More flexible materials were compared with Co-Cr to optimize the stress on the abutment in FES RPD scenarios such as gold alloys, titanium alloys, stainless clasps, PEEK, and the use of resin clasps (polyesters and polyamides) [62–65]. PEEK was proven as an attractive option to replace the Co-Cr due to the minimum stress on the PL of the abutments and at the same time showed adequate retention [63, 66]. To optimize the retention of the PEEK clasp in a 0.01-inch undercut, the ratio width/thickness at the tip shall be 1.50/1.13, 1.75/1.53, or 2.70/1.69 [63].

Rigid and nonrigid attachments are considered efficient retainers to FES RPD with no visible metal components. Use of nonrigid attachment results in less stress in the main abutment but on the other hand brings more stress to the supporting ridge. The concentration of the stress in the ridge was obvious in the mesial and distal area of the saddle [67].

#### 3.3.2. Occlusal Rest Position

The occlusal rest position shows a role in stress distribution in abutment teeth and RPD framework. Putting the occlusal rest on the distal side of the terminal abutment was found to improve the stress distribution in these teeth and stiffen the metal frameworks and acrylic resin denture bases by 66% when compared with the occlusal rest placed on the mesial side of the same abutment [68].

#### 3.3.3. Design of Major Connectors

While the retentive arm of retainers should be fabricated from flexible materials, major connectors should be rigid to provide an equal distribution of load and prevent stress concentration in supporting structures. The prosthesis with a highly rigid major connector is associated with less deflection during function [69]. The deflection of the prosthesis results in unequal distribution of the stress in the underlying structures, which leads to inflammation and resorption of the residual ridge [69]. The stress developed in supporting structures depends on the material of fabrication, design and thickness of the used major connector, and the shape of the palate [66]. The anteroposterior palatal strap design was found to be the most rigid design compared with different designs such as complete palatal plate, posterior palatal strap, and, lastly, horseshoe-shaped major connector, which showed the lowest rigidity [69, 70]. To increase the rigidity and to reduce the internal stres in of horseshoe-shaped major connectors, a double thickness was advocated. This modification, however, can deliver higher stress on the underlying mucosae and PL [71]. The shape of the palate also influences the stress and displacement of the major connector [72, 73]. The narrow palate shows the least displacement in the major connector comparing the wide and shallow palate [72, 73]. The flexible framework materials are always associated with less stress in the major connector, but more displacement on the ridge is recorded [66, 73, 74].  Table 3 shows that the studies evaluated the influences of major connectors on the stress and deflection of RPDs.  Table 3 shows also the lack of literature in evaluation of stress developed in the different designs of mandibular major connectors.

#### 3.3.4. Splinting of the Abutment Teeth

Teeth with reduced periodontal support are considered inadequate abutments for retention and support of RPDs, especially RPDs with distal extension scenarios. However, splinting two or more reduced periodontally supported teeth was beneficial for adequate stress distribution and reduction of the anterior displacement of these teeth. Splinting more than three teeth around the arch was more beneficial as it can provide a curve for resistance to the buccolingual displacement [75].

#### 3.3.5. Use of the Implant Approach

Implant-assisted RPDs were advocated for FES scenarios to provide a substantial increase in retention of the RPD as well as reduction of the stress in the abutments and residual ridges. According to the implant-assisted RPD concept, the implant and surrounding bone (especially the cancellous) receive the majority of the stress, while abutments receive minimal stress and the displacement of the prosthesis becomes minimal [21, 47–49]. On the other hand, the acrylic base of the prosthesis over the abutment of the implant receives a significant amount of stress, which may lead to the frequent fractures of this part of acrylic around the attachment. This is mainly due to the mismatch of the distribution of stress between the acrylic bases and the base metal framework as the stresses developed in the metal frameworks mainly concentrated in the major and minor connectors away from the attachment area, while the occlusal load transfers directly to the acrylic materials around the attachment [49].

The stress developed on the abutment, implants, and denture base materials varies according to the implant location, inclination, diameter, and type of applied load.  Table 4 shows the influences of implant designs on the development of stress and displacement.

### 3.4. Factors Affecting Stress Developed in Implant-Assisted RPDs

#### 3.4.1. Implant Location

Implant locations were found to have direct influences on the development of stress in abutments and residual ridge. When the implant is placed more anteriorly (the premolar area), the stress on the implant became maximum, the stress on the abutment teeth became minimum, and the displacement distally became maximum. On the other hand, when the implant is placed more posteriorly (the 2^nd^ molar area), the stress on the abutment teeth becomes considerably high and more displacement is reported at the mesial side of the residual ridge. Placement of the implant in the first molar area has been proven to have the lowest stress in the implant, lowest stress in abutment teeth, and lowest stress in the distal side of the residual ridge [17, 55, 76–79].

#### 3.4.2. Length and Diameter of the Implant

The length and diameter of the implant influence the displacement and stress in abutment teeth, denture supporting structures, and bone surrounding the implant. Long implants were found to decrease the stresses in abutments and minimize the stress in the surrounding bone, especially the cancellous one [81]. Similarly, the wide implants were found to decrease the displacement of the prostheses, decrease the stresses in abutments, and minimize the stress in both cortical and cancellous bone [79].

#### 3.4.3. Implant Angulation

Different angulations of implants were evaluated to estimate their influences on the development of stress in abutments and implants [81, 82]. The inclinations of the implant result in the improvement of stress in abutment teeth but increase it on the bone surrounding the implant to reach its maximum extent with angulation of 15° [81] and 30° [82].

Even though the FEA has been used in the estimation of stresses in RPDs for 20 years, still lack of FEA studies in the literature exists. The lack is mainly regarding the estimation of stresses in different designs of RPDs (especially the bounded and anterior edentulism), different designs of mandibular major connectors, use of short and narrow implants, and use of different systems of attachments.

## 4. Conclusion

Within the limitation of the present study, the following can be concluded:

1. Implants in implant-assisted RPDs receive the majority of the dynamic load. The magnitude of the load decreases with zero or minimal angulations of the implant, using long and wide implants, and when the implant is placed in the first molar area
2. Stress in FES RPDs are concentrated in the shoulder of the clasp, the minor connector of the mesial rest, and the part of major connector next to the terminal abutment
3. Clasps with flexible arms decrease the stress in abutment teeth, while the rigid major connector decreases the displacement and stresses in the residual ridge
4. The distal occlusal rest stiffens the framework and decreases the stress on the terminal abutment
5. Resilient attachments put less stress in abutments but increase the stress in the residual ridge, especially the posterior part of the saddle
6. A lack of FEA studies covering many aspects of different designs of RPDs exists

## Figures and Tables

**Table 1:** Inclusion and exclusion criteria.

Inclusion criteria	Exclusion criteria
1. Only studies that used FEA	1. Experimental and *in vivo* studies
2. Full-text studies	2. Letter to editor or conference studies
3. From Jan 2000 to May 2021	3. Before Jan 2000 or after May 2021
4. Only in English or translated papers	4. Other than English or not translated studies
5. Only studies conducted on RPD	5. The studies conducted in removable complete or fixed restoration

**Table 2:** Studies reporting the influence of different retainer designs on stress and displacement of conventional RPD.

Authors	Type of prosthesis	The examined independent variable	Materials used in the denture	Reported dependent variable		Outcome
				Stress	Displacement deflection	
Richert et al. 2021 [60]	FES RPD	Optimizing the length of I-bar clasp	Co-Cr	√	—	(i) I-bar clasp design could demonstrate optimal mechanical properties as long as the length of horizontal and vertical arms did not exceed 6 mm length
Tribst et al. 2020 [55]	BS RPD	18 3D designs of Aker clasps, with different materials, within 0.25, 0.5, and 0.75 mm undercuts	Six materials: (i) Polyamide (ii) Polyoxymethylene (iii) PEEK (iv) Gold alloy (v) Titanium (Ti–6Al–7Nb) Co-Cr	√	√ and force of removal	(i) The stress was concentrated at the shoulder of the circumferential clasp in all models (ii) The highest stress was reported in Co-Cr with 0.75 mm, while the lowest stress was reported in polyamide, regardless of depths of undercuts (iii) Polyamide showed the lowest forces of removal, followed by polyoxymethylene, while Co-Cr showed the highest removal force followed by titanium
Peng et al. 2020 [63]	—	72 3D models of PEEK clasps with different thickness/width ratios	(i) PEEK (ii) Co-Cr	√	√ and force of removal	(i) The maximum stress concentration was located at the base of the clasp (ii) The stress concentration increased when the thickness of the material increased (iii) PEEK clasp showed higher flexibility when compared with Co-Cr clasp (iv) PEEK clasp with a ratio of width/thickness at the tip 2.70/1.69, 1.50/1.13, or 1.75/1.53 was considered an optimal clasp to 0.5 mm undercut
Yamazaki et al. 2019 [64]	Mandibular FES RPD	Resin clasp with 6 areas of blocked-out undercut with 0.50 & 0.75 mm on the buccal surface of the main abutment	Co-Cr base denture with two thermoplastic resin clasps (i) Polyester polyamide	√	√ and force of removal	(i) The stress was concentrated at the shoulder of the clasps but on the inner surface (ii) No significant differences were reported between the two types of resin (iii) The retention of thermoplastic clasps depends on the position and depth of undercut rather than the material itself
Reddy et al. 2016 [57]	Mandibular BS RPD	2 Aker clasps, with two different materials, in 0.25 mm undercut	(i) Co-Cr (ii) Acetal resin	√	√ and force of removal	(i) The highest stress was reported in the Co-Cr clasp compared with the acetal resin (ii) The force of removal of acetal resin was significantly lesser than that of the Co-Cr
Nakamura et al. 2014 [58]	Mandibular FES RPD	(i) 1 Aker and 1 reverse Aker (ii) 1 embrasure clasp (iii) 1 I-bar clasp	Co-Cr	√	√	(i) RPI clasp shows lower stress concentration in the buccal and apical region and areas of the cortical bone supporting the abutment tooth when compared with Aker and embrasure clasps (ii) Embrasure clasp expressed slightly lesser vertical displacement compared with RPI and Aker clasps, while RPI showed significantly lesser distal displacement followed by embrasure and Aker clasps
Oyar et al. 2012 [44]	—	9 3D models of the I-bar clasp of three different materials and three modified tips	(i) Co-Cr (ii) Titanium (Ti–6Al–7Nb) (iii) Gold alloy	√	—	(i) The maximum stress concentration was located at the horizontal curvature of the clasp and was reported in the Co-Cr specimen, while the gold alloy specimen showed the minimum stress (ii) There is a direct relationship between lengths of the horizontal arm and development of stresses in the arms of the clasp
Wang et al. 2011 [67]	Mandibular class II	Rigid and nonrigid precision attachment (ERA attachment)	Ni-Cr	√	—	(i) Both attachments showed similar stress distribution in the alveolar bone and PL, but with more concentration in the case of rigid attachment (ii) Compared with the rigid attachment, the nonrigid attachment resulted in higher stress in the mesial and distal end of the residual ridge when subjected to axial loads; however, the opposite was true regarding buccolingual and mesiodistal loads
Aoda et al. 2010 [59]	Mandibular FES RPD	(i) Reverse Aker (ii) Embrasure (iii) Back action	Co-Cr	√	√	(i) Reverse Aker clasp put more stress in abutment teeth compared with embrasure and back action clasps (ii) Reverse Aker provided higher stability and lesser deflection to the denture compared with embrasure and back action clasps
Sandu et al. 2010 [62]	Maxillary FES RPD	Evaluation of round and half-round clasps with 9 diameters (from 0.5 to 1.3 mm) for each	Stainless steel	√	√	(i) The stress was concentrated in the inner surface of both half-round and round wires, in the part of the arm located above the height of contour of abutment teeth (ii) Regarding the displacement, the clasp arm with half-round shape (with a diameter of 1 mm) showed a similar displacement to the clasp arm with round shape (with a diameter between 0.6 and 0.7 mm)
Judy 2009 [56]	FES RPD	Optimizing the width & length of the circumferential clasp arm	Co-Cr	√	—	The circumferential clasp with half-round shape and formula W2/W1 = 0.6 and *T*/*L* = 0.5 showed the least stress concentration
Sato et al. 2001 [61]	FES RPD	Evaluation of the I-bar clasp with 6 widths & lengths	Co-Cr	√	√	(i) I-bar clasp with thin and wide arm, taper 0.020-0.023, and radius of curvature of 2.75–3.00 exhibited less stress compared with the thicker or shorter ones

FES: free-end saddle; BS: bounded saddle; Co-Cr: cobalt-chromium; Ni-Cr: nickel chromium; PEEK: polyetheretherketone; W2: the width of the clasp at the tip; W1: the width of the clasp at the base; T: thickness; L: length; PL: periodontal ligaments.

**Table 3:** Studies reporting the influence of different designs of the major connector on the stress and displacement of conventional RPD.

Authors	Type of prosthesis	The examined independent variable	Materials used in the denture	Reported dependent variable		Outcome
				Stress	Displacement deflection	
Rodrigues et al. 2021 [74]	Maxillary class I	Two 3D models of two different materials	Two materials (i) Co-Cr (ii) Thermoplastic nylon (flexible denture)	√	√	(i) In both models, the maximum stress has been shown on the slopes of the maxillary arch (ii) The maximum displacement has been shown on the crest of the residual alveolar ridge (iii) The Co-Cr showed the least stress and displacement compared with nylon
Chen et al. 2019 [66]	Mandibular class I	Three models for three different materials	3 materials (i) Co-Cr (ii) Ti alloy (iii) PEEK			(i) The lowest stress in the PDL of the abutment and framework was reported with PEEK (ii) PEEK has exhibited the highest displacement of the ridge and mucosa
Hallikerimath et al. 2015 [72]	Maxillary class II RPD	Five 3D models of different palatal vaults (average, wide, narrow, deep, and shallow)	Co-Cr	—	√	(i) The maximum distal displacement was reported in the wide and shallow palate, while maximum buccal displacements were higher in the deep palate (ii) Maximum vertical displacement was higher in the average model (iii)The deflection was lesser in the narrow palate compared to the other palatal shapes
Bhojaraju et al. 2014 [69]	Different scenarios of maxillary RPD	Six 3D models of 3 different maxillary MC (PS, CPP, APPS) with different scenarios of Kennedy classification	Co-Cr	—	√	(i) APPS showed the lowest deflection compared with CPP and PS (ii) For APPS, the maximum deflection was reported in the occlusal rest responding to load with anteroposterior direction and the anterior part of buccal slope regarding vertical direction (iii) For CPP, the maximum deflection has been reported in the occlusal rest regarding anteroposterior load and the buccal slope and crest of the ridge regarding vertical force
Ramakrishnan & Singh 2010 [71]	Maxillary class IV	Four 3D models of U-shape PB (regular, increasing the width, adding posterior PS, and duplicating the thickness to 1 mm)	Co-Cr	√	√	(i) The PB with a regular width showed the maximum deflection and displacement compared with the other forms (ii) The double-thickness U-shape MC exhibited the lowest stress followed by wide U-shape MC (iii) The highest stress on the palate and teeth has been shown in double thickness as well (iv) The lowest stress on the palate and mucosa has been reported in the scenario of wide MC
Takanashi et al. 2009 [73]	Maxillary class II	Five 3D models of different palatal vaults (basic, wide, narrow, deep, and shallow)	Three materials were used: (i) Co-Cr (ii) Titanium (Ti–6Al–7Nb) (iii) Gold alloy (type IV)	—	√	(i) In all tested MC models, the narrow model has reported the lowest displacement when compared with the basic, wide, and shallow palates, which exhibited the maximum displacement (ii) In the deep palate model, the Ti MC with a width of 11 mm and gold MC with a width of 9 mm showed similar displacement to the basic model
Eto et al. 2002 [70]	Maxillary class II RPD	In 13 3D models, 11 of them show PS MC with different AP widths at the midlines, 1 design for APPB, and lastly, horseshoe PS with 7 mm	Co-Cr	—	√	(i) The maximum displacement has been shown in all models at the posterior edge of the saddle (ii) Vertical and buccal displacements were inversely proportional to the width of the major connector. As the major connector increased, the displacement decreased (iii) APPB and wide PS exhibited the lowest buccal displacement compared with horseshoes, which showed the maximum displacement (least rigidity)

FES: free-end saddle; AP: anteroposterior; PS: palatal strap; APPS: anteroposterior palatal strap; APPB: anteroposterior palatal bar; CPP: complete palatal plate; MC: major connector; Ti: titanium.

**Table 4:** Studies reporting the biomechanical stress and displacement in implant-assisted removable partial denture with different designs.

Authors	Type of prosthesis	The examined independent variable				The reported dependent variable			Outcome
		Length	Width	Location	Inclination	Attachment	Stress	Deflection	
Tribst et al. 2020 [55]	Four 3D models of conventional and ISRPD class II mod 2 with 3 different designs	—	—	(i) M1 at the 1^st^ molar (ii) M2 at the 2^nd^ molar (iii) M3 2 implants at the 1^st^ and 2^nd^ molars					(i) The highest stress concentration in the implant has been reported in the implants of M2 followed by M3 (ii) The implant in the 1^st^ molar region received less stress as in M1 and M3
Messias et al. 2019 [76]	Two 3D models of mandibular class I IARPD in 2 different locations	—	—	(i) M1 implants located at the premolar area (ii) M2 at the premolar area	—	—	√	—	(i) The implant located in the premolar area exhibited the highest displacement in the posterior region, while the opposite happened when the implant was located in the molar area (ii) The stress was more concentrated in the part of the major connector next to abutment teeth (iii) More stress on the posterior part of the saddle was shown when the implant was located in the premolar area
Ortiz-Puigpelat et al. 2019 [77]	Two 3D models of mandibular class I IARPD in 2 different locations	—	—	Three different locations (i) M1 at the 2^nd^ molar (ii) M2 at the 1^st^ molar (iii) M3 at the 2^nd^ premolar	—	—	√	—	When the implant was located in the 1st molar area, less displacement and minimum stress at the implant and the metal framework were reported
Andrei et al. 2015 [43]	One model for conventional and IARPD for Co-Cr mandibular class I	—	—	2 implants were placed bilaterally in the 2^nd^ molar area	—	—	√	√	(i) In the conventional RPD, the maximum stress was reported at the anterior (premolar) and posterior (2^nd^ molar) areas (ii) There was a reduction in the maximum stress at the same area in IARPD compared with the conventional RPD (iii) The lateral displacement was high at the distal edge of both prostheses but with a higher value in the conventional RPD than in IARPD
Memari et al. 2014 [17]	Three 3D models, one for class II IARPD in 3 different locations	—	—	(i) M1 at the 2^nd^ molar area (ii) M2 at the 1^st^ molar area (iii) M3 at the 2^nd^ premolar area	—	—	√	—	As the implant was placed more anteriorly, more stress was concentrated in the terminal abutment, reaching its maximum value when the implant was located next to the terminal abutment
Cunha et al. 2008 [80]	Five models: (i) Natural (ii) Conventional (iii) IARPD (3 models) Co-Cr mandibular class II	—	—	The implant location: (i) Distal (2nd molar) (ii) Middle (1st molar) (iii) Mesial (1st premolar)	—	—	√	√	(i) In all IARPD designs, there was a clear diminish in the displacement when compared with the conventional RPD (ii) In IARPD, the lowest displacement has been exhibited in an implant located in the middle of the residual ridge and then the distal area of the ridge, while the mesial location of the implant showed the lowest stress in the terminal abutment (iii) The mesially placed implant showed the highest stress value in the internal thread of the implant followed by the middle and then the distal area, which showed the least stress
El-Okel and Elnady 2013 [79]	Six 2D models (i) Natural (ii) Conventional (iii) IARPD (4 models) Co-Cr mandibular class II	10 mm	3 & 3.5 mm	One implant in the 6^th^ and one implant in the 7^th^ molar region	—	—	√	—	(i) The stress on the terminal abutment was the least with an implant of 10 × 3.5 and then 10 × 3 in the 1st molar area, compared with implants of 10 × 3.5 and 10 × 3, at the 2nd molar area (ii) The highest stress has been recorded in the implant of 10 × 3 and located in the 2^nd^ molar area, while the lowest stress has been recorded in the implant of 10 × 3 and located in the 1st molar area
Fayaz 2015 [81]	Six 3D models of IARPD with two lengths and 3 different inclinations	(i) M1-3 (7 mm) M4-6 (10 mm)	4 mm	1^st^ molar area	(i) M1-3 at 0°, 10°,and 15° M4-6 at 0°, 10°, and 15°, respectively	—	√	—	(i) Increasing the inclination of the implant has shown increase in the stress in the implant to reach the maximum in M6, while the stress in the terminal abutment decreased to the minimum (ii) As the length of the implant increased, the stress on the abutment decreased
de Freitas Santos et al. 2011 [82]	Six 3D models of natural, conventional, and IARPD mandibular class II with 4 different angles	—	—	2^nd^ molar region	4 models with different inclinations were used: (0°, 5°, 15°, and 30°) in a mesial direction	—	√	√	(i) Adding an implant to assist RPD led to a significant reduction in the displacement of the prostheses (ii) The stress around the apex of the terminal abutment in all models with implants has shown better distribution in 0° and 5° compared to 15° and 30°, which showed the highest stress

M: model; IARPD: implant-assisted removable partial denture.

## Data Availability

All data are available within the manuscript.
